# COVID‐19 and ENT SLT services, workforce and research in the UK: A discussion paper

**DOI:** 10.1111/1460-6984.12565

**Published:** 2020-08-08

**Authors:** Joanne M Patterson, Roganie Govender, Justin Roe, Gemma Clunie, Jennifer Murphy, Grainne Brady, Jemma Haines, Anna White, Paul Carding

**Affiliations:** ^1^ Liverpool Head and Neck Centre/School for Health Sciences University of Liverpool, Liverpool UK; ^2^ Head and Neck Cancer Centre and Research Department of Behavioural Science & Health University College Hospitals London University College London London UK; ^3^ National Centre for Airway Reconstruction Department of Otolaryngology, Head and Neck Surgery Imperial College Healthcare NHS Trust London UK; ^4^ Department of Surgery and Cancer Imperial College London UK; ^5^ Department of Speech, Voice and Swallowing The Royal Marsden NHS Foundation Trust London UK; ^6^ Department of Speech, Voice and Swallowing, ENT Outpatients Newcastle Upon Tyne Hospitals NHS Trust Newcastle Upon Tyne UK; ^7^ Wythenshawe Hospital Manchester University NHS Foundation Trust University of Manchester NIHR Manchester Biomedical Research Centre Northwest Lung Centre Manchester UK; ^8^ Department of Ear, Nose and Throat, Queens Medical Centre Nottingham University Hospitals NHS Trust Nottingham UK; ^9^ Oxford Institute of Nursing, Midwifery and Allied Health Research Faculty of Health & Life Sciences Oxford Brookes University Oxford UK

**Keywords:** COVID‐19, ENT SLT services, ENT SLT workforce, ENT SLT research

## Abstract

**Background:**

The COVID‐19 pandemic and the UK government's subsequent coronavirus action plan have fundamentally impacted on every aspect of healthcare. One area that is severely affected is ear, nose and throat (ENT)/laryngology where speech and language therapists (SLTs) engage in a diverse range of practice with patients with a range of conditions, including voice disorders, airway problems, and head and neck cancers (HNCs). A large majority of these patients are in high‐risk categories, and many specialized clinical practices are vulnerable. In addition, workforce and research issues are challenged in both the immediate context and the future.

**Aims:**

To discuss the threats and opportunities from the COVID‐19 pandemic for SLTs in ENT/laryngology with specific reference to clinical practice, workforce and research leadership.

**Methods & Procedures:**

The relevant sections of the World Health Organisation's (WHO) health systems building blocks framework (2007) were used to structure the study. Expert agreement was determined by an iterative process of multiple‐group discussions, the use of all recent relevant policy documentation, and other literature and shared documentation/writing. The final paper was verified and agreed by all authors.

**Main Contribution:**

The main threats to ENT/laryngology SLT clinical services include increased patient complexity related to COVID‐19 voice and airway problems, delayed HNC diagnosis, reduced access to instrumental procedures and inequitable care provision. The main clinical opportunities include the potential for new modes of service delivery and collaborations, and harnessing SLT expertise in non‐instrumental assessment. There are several workforce issues, including redeployment (and impact on current services), training implications and psychological impact on staff. Workforce opportunities exist for service innovation and potential extended ENT/SLT practice roles. Research is threatened by a reduction in immediate funding calls and high competition. Current research is affected by very limited access to participants and the ability to conduct face‐to‐face and instrumental assessments. However, research opportunities may result in greater collaboration, and changes in service delivery necessitate robust investigation and evaluation. A new national set of research priorities is likely to emerge.

**Conclusions & Implications:**

The immediate impact of the pandemic has resulted in major disruption to all aspects of clinical delivery, workforce and research for ENT/laryngology SLT. It is unclear when any of these areas will resume operations and whether permanent changes to clinical practice, professional remits and research priorities will follow. However, significant opportunity exists in the post‐COVID era to re‐evaluate current practice, embrace opportunities and evaluate new ways of working.

**What this paper adds**

**What is already known on the subject**

- ENT/laryngology SLTs manage patients with a range of conditions, including voice disorders, airway problems and HNCs. The diverse scope of clinical practice involves highly specialized assessment and treatment practices in patients in high‐risk categories. A large majority of active research projects in this field are patient focused and involve instrumental assessment. The COVID‐19 pandemic has created both opportunities and threats for ENT SLT clinical services, workforce and research.

**What this paper adds to existing knowledge**

- This study provides a discussion of the threats and opportunities from the COVID‐19 pandemic for ENT/laryngology SLT with specific reference to clinical practice, workforce and research leadership.

**What are the potential or actual clinical implications of this work?**

- The COVID‐19 pandemic has resulted in major disruption to all aspects of clinical delivery, workforce and research for ENT/laryngology SLT. Changes to clinical practice, professional remits and research priorities are of indeterminant duration at this time, and some components could be permanent. Significant clinical practice, workforce and research opportunities may exist in the post‐COVID era.

## Introduction

The UK government's coronavirus action plan to the COVID‐19 pandemic includes four stages: containment, delay, research and mitigation. The consequent legislation (The Health Protection Regulations 2020) enables action in five main areas: (1) increasing the available health and social care workforce; (2) easing the burden on frontline staff and prioritizing care for people with the most pressing needs; (3) containing and slowing the virus by reducing unnecessary social contacts: (4) managing the deceased with respect and dignity; and (5) supporting people. These measures are temporary and subject to regular review. However, consequently, the delivery of every aspect of healthcare by all clinical and non‐clinical departments in the National Health Service (NHS) has been fundamentally affected (Willan *et al*. 2020). The Royal College of Speech and Language Therapists (RCSLT) has stated that, as specialists in communication and swallowing, speech and language therapists (SLTs) have a crucial role to play as part of wider multidisciplinary teams (MDTs) in this response (RCSLT 2020a). This includes: (1) supporting people with communication and swallowing needs who contract the virus; (2) ensuring relevant information is accessible to those who have speech, language and communication needs; (3) urging relevant retirees and graduates to increase the available health and social care workforce; and (4) reducing risk of contracting the virus to SLTs, their loved ones and those they care for and work with.

The NIHR CRN ENT SLT (National Institute for Health Research Clinical Research Network, Ear Nose and Throat sub‐specialty Speech and Language Therapy) Research Group represents a collaboration of experienced clinical researchers in ENT/laryngology (voice and airway disorders, and head and neck cancer—HNC). This group provides a broad geographical representation of specialist ENT/laryngology clinical services in England and includes all the main researchers in the field. A large majority of this group are also at the forefront of clinician services associated with COVID‐19 NHS service delivery.

ENT SLT services have seen major alterations during the pandemic. Direct changes include a steep increase in referrals for COVID‐19‐related voice, swallowing and airway problems. Indirectly, voice, airways and HNC patients have experienced less face‐to‐face SLT contact, more remote consultations, fewer joint clinics and less access to instrumental assessments, with alterations and delays to their proposed treatment. The aim of this paper is to describe these changes and potential consequences in more detail, and to discuss the threats and opportunities from the COVID‐19 pandemic for SLTs in ENT/laryngology practice.

## Methodology

This discussion paper represents the consensus view of the NIHR CRN ENT SLT Research Group (see the acknowledgements). A series of discussions has been distilled by the authors and verified by the whole group before publication. We have used the World Health Organisation's (WHO) health systems building‐blocks framework (WHO 2007) to select those relevant to this discussion, namely: service delivery (clinical consideration), workforce and leadership/governance (research implications). The expert consensus of the current context and the future implications and opportunities are discussed for each of these three areas.

## Service delivery/clinical implications

ENT SLTs engage in a diverse scope of clinical practice with many working within highly specialized MDTs. These teams serve patients with a range of conditions including HNCs, benign voice disorders and airway problems. Many of these patients fall into the ‘vulnerable’ category, in particular those with a laryngectomy or long‐term tracheostomy, who have an increased risk of pneumonia (El Cheikh *et al*. 2018).

The pandemic presents new clinical challenges for the foreseeable future with 95.6% of respondents in a recent RCSLT survey stating that the pandemic was having an impact on their professional roles, responsibilities and duties (RCSLT 2020b). Here we summarize the impact of COVID‐19 on: (1) existing ENT SLT services and service delivery, before highlighting clinical considerations, adaptations and potentially new clinical opportunities for each of three main patient groups within this sphere of practice: (2) HNC services, (3) voice services and (4) airways services.

### General ENT SLT clinical implications

Under usual circumstances professional guidelines advise that all patients with suspected laryngeal disorders should undergo endoscopic evaluation of the larynx (EEL) (RCSLT 2019). Similarly, endoscopic or videofluoroscopic evaluations of swallowing are key diagnostic and therapeutic techniques. These specialized services have been disrupted by the pandemic with prioritization of resources, rigorous risk criteria and new protocols being introduced to limit virus transmission (RCSLT 2020c, 2020d). This cessation of routine nasendoscopic procedures impacts patient care with the potential for misdiagnosis, suboptimum treatment and unnecessary healthcare utilization (Idrees and FitzGerald 2015).

In the absence of endoscopy ENT SLTs are using their clinical skills for the benefit of patients. ENT SLTs can assess and manage patients using detailed and thorough history‐taking, perceptual and acoustic evaluation of voice and comprehensive clinical assessment of swallowing. They are also able to use their work with patients to prioritize those who do need further instrumental evaluation.

Routine, face‐to‐face SLT outpatient appointments have been postponed indefinitely to limit unnecessary footfall in hospitals with 75% of SLTs reporting that there are patients on their caseload no longer receiving intervention when they would usually do so (RCSLT 2020b). Many SLT procedures, including dysphagia assessment, have been designated high‐risk Aerosol Generating Procedures (AGPs) in the RCSLT professional guidelines and evidence review (Bolton *et al*. 2020). This means that emergency cases such as urgent laryngectomy voice prosthesis changes require ENT SLTs to follow rigorous infection control guidance and wear full personal protective equipment (PPE).

Innovative approaches to continuing and re‐establishing clinical practice are being trialled and implemented. Over 70% of SLTs surveyed have identified changes made as a result of the pandemic that are of benefit to their clinical practice, patients and/or service (RCSLT 2020b). This has included setting up telehealth services to offer assessment and rehabilitation via telephone and video‐conferencing reviews. This has benefits to patients with previous research showing favourable clinical outcomes and good acceptance (Burns *et al*. 2017). The potential for aspects of telehealth practice to be incorporated within the ENT SLT pathway beyond the pandemic is already part of the conversations amongst many teams.

Another challenge with the interruption in outpatient ENT SLTs has been the reduction in MDT clinics. Close liaison with surgical, allied health and nursing colleagues is crucial to form a clear, well‐evidenced treatment plan for ENT patients. Limitations to face‐to‐face MDT contacts have been overcome using telehealth and forward planning for services to reopen in the most efficient, safe and effective way possible for patients.

For ENT SLTs there are very real concerns that the current climate means that people are delaying general practitioner and accident and emergency (A&E) visits with health concerns (*NHS England News* 2020). If patients present with later stage (more advanced) disease, they will require more intensive treatment, and by extension, greater SLT rehabilitation needs. Undiagnosed laryngeal disorders might worsen over time, increasing their complexity and hindering treatment response (Stachler *et al*. 2018).

ENT SLTs also need to be vigilant of the high prevalence of laryngeal injury post‐extubation in intensive care unit (ICU) settings, which can cause dysphonia and/or dysphagia (Brodsky *et al*. 2018) Laryngeal trauma might also arise from acute cough (Slinger *et al*. 2019), with post‐viral symptoms impacting voice and swallowing even in patients who have not been admitted to hospital. This leads to the potential for a surge of new referrals for ENT SLTs as the initial peak of the pandemic passes and needs to be accounted for in service delivery.

### Head and neck cancer (HNC) services

In the UK NHS, HNC is managed by MDTs working within cancer centres, with the SLT being a core MDT member (National Institute for Health and Clinical Excellence 2004). As such, SLTs are involved at every stage of an individual's care from diagnosis and pre‐habilitation throughout oncological treatment, post‐treatment rehabilitation, survivorship and palliation. The current pandemic has triggered a range of changes to many aspects of the hitherto relatively well‐established head and neck patient pathway.

#### New referral pathways

Most suspected cancer referrals are received via the 2‐week wait referral pathway (Department of Health 2000) via general practitioners or dentists, or through A&E departments. This referral rate has significantly reduced due to public fears in accessing healthcare.

For referrals that are received via a 2‐week wait referral pathway, hospital consultants are using telephone consultations. Without the benefit of face‐to‐face clinical examination, alternate innovations that enhance differential diagnosis is vital. One such innovation is the use of a remote evidence‐based triage system (Paleri *et al*. 2020) to stratify patients according to likely risk of cancer. Patients with voice and swallowing symptoms, which are least likely to be attributable to a cancer diagnosis, could potentially be directed toward SLT‐led clinics (see voice services below).

#### MDT meetings and clinics

Weekly MDT meetings are a feature of all cancer centres in the UK and are the focal point for discussion and decision‐making around each patient's individual treatment plan. These meetings are now either severely reduced or conducted virtually. SLT input around functional outcome in relation to different treatment options is compromised. Instead, discussions are often abridged with a focus on which patients require urgent treatment, who can be delayed and how to best provide oncological treatment with least risk to patients and staff. Relevant professional bodies have issued guidance and collaborative statements around management of HNC patients during the pandemic (ENT‐UK 2020), which temporarily replace existing guidelines (National Institute for Health and Clinical Excellence 2004). SLTs may experience loss of professional identity within the MDT, but more importantly delays to clinical input, decision‐making as well as losing the benefit of joint working may have negative consequences for patients. Patients may encounter mixed messages from the team that can add confusion and anxiety to an already stressful event of receiving a cancer diagnosis. This may escalate into negative consequences for building trust with the team and could potentially influence patient engagement and subsequent outcomes.

#### Cancer treatment regimes

The surgical and chemo/radiotherapy treatment regimes for HNC have been adjusted in response to the risks associated with coronavirus transmission (Day *et al*. 2020). Accordingly, the British Association of Head and Neck Oncologists (BAHNO) have issued treatment decision guidance during the pandemic (BAHNO 2020). They recommend avoidance of laryngectomy procedures unless considered absolutely necessary and to treat with radiotherapy instead. However, voice and swallowing can be adversely affected in patients with advanced laryngo‐pharyngeal cancer treated with radiotherapy (Rosenthal *et al*. 2015). Furthermore, treating residual or recurrent disease with salvage laryngectomy can lead to poorer voice and swallowing outcome (Burnip *et al*. 2013). Where primary laryngectomy is performed, tracheoesophageal puncture (TEP) should be avoided to reduce the risk of early postoperative complications. Although a secondary TEP can be performed later, SLTs will need to prepare and rehabilitate patients using non‐surgical voice‐restoration methods. Access to appropriate communication aids such as electrolarynx devices is therefore imperative.

BAHNO has also advised limiting operation times wherever possible, for example, use of local flaps instead of free flaps. Minimally invasive surgery is currently not accessible (Day *et al*. 2020). This has implications for functional outcome. For some, superior speech and swallowing can be achieved with local flaps, where structures are mobile and tethering is limited (Lam and Samman 2013). However, large defects insufficiently reconstructed can result in sumps and reduced proximity of anatomical structures, affecting articulation accuracy and pressure generation for efficient and safe swallowing (Lam and Samman 2013). Furthermore, BAHNO have advised restricting the use of chemoradiotherapy, and treating solely with radiotherapy, wherever possible. This alteration to traditional treatment regimes may result in superior outcomes for swallowing as single modality treatments tend to be less deleterious for functional outcomes (Wilson *et al*. 2011). These adaptations to treatment have implications for the pretreatment discussions SLTs have with patients and their expectations for their subsequent function and rehabilitation.

It is also important to highlight that changes in treatment regimes during the pandemic mean that many patients will not receive the optimum treatment plan they may have otherwise been offered. The emotional and psychological response that individuals may have to this cannot be underestimated and may be an important area for further research.

#### Rehabilitation

Clinicians have had to be innovative in managing their HNC outpatient caseloads. Whilst there is likely to be much variation throughout the country, it is encouraging to note that large numbers SLT services have proactively adapted to provide rehabilitation via telephone (60.7%) and video‐conferencing (43.6%) while promoting self‐management where possible (RCSLT 2020b). However, not all patients are suitable or indeed receptive to telehealth, and the ramifications of delaying rehabilitation will need to be carefully considered as we move through the pandemic and decide on priorities for restarting face‐to‐face services. RCSLT clinical excellence networks (CENs) have played an important part in disseminating and sharing experiences via social media and through the CEN websites. Patient organizations such as the National Association of Laryngectomy Clubs and the Swallows Charity amongst others have also played an important supportive role for both patients and carers alike.

### Voice services

#### Referral patterns

Referral patterns to voice and upper airway services will depend upon the activity of existing referral sources who may be seeing fewer patients, in conjunction with anticipated increased demands from COVID‐19 patients. New referral sources might include respiratory medicine and intubation trauma services. Notably, the newly proposed remote evidence‐based triage system described above (Paleri *et al*. 2020) could further increase referrals for SLT‐led EEL clinics. An ENT SLT‐led Two Week Wait HNC referral pathway for low risk hoarse patients pilot study demonstrated an excellent safety record for cancer screening and more efficient access to voice services (Slade and McGlashan 2019). This role extension requires clear protocols, parallel consultant ENT input and Trust‐level clinical governance clearance. Delays to elective phonosurgery, including injection laryngoplasty, may also demand intermediate voice therapy and/or provision of amplification devices (White 2019). Postponement of such treatments could have huge psychosocial implications for patients, particularly those who are socially isolating, as telephone use is a common challenge for individuals with dysphonia.

#### Clinical implications of COVID‐19

Post‐extubation injuries can include laryngeal oedema, arytenoid dislocation, laryngeal ulceration and vocal cord paralysis (Brodsky *et al*. 2018, McGrath *et al*. 2020). Notably, intubation can cause superficial mucosal damage and stiffness, affecting vocal fold pliability required for voicing (Hirano and Kakita 1985). Implications of such disorders can pose short‐ and long‐term challenges to communication, at a time when speaking with loved ones and healthcare professionals is essential for well‐being and enabling joint decision‐making to promote patient‐centred care.

For those diagnosed with mild to moderate COVID‐19, 27% reported dysphonia, and was more common in females and smokers (Lechien *et al*. 2020). Time to recovery is as yet unreported.

Hyperfunctional voice/laryngeal disorders might also present secondary to proliferate use of telecommunications for work and social interaction, speaking through PPE and/or through increased levels of anxiety (Besser *et al*. 2020). A ‘vicious cycle’ may arise between anxieties and laryngeal function, particularly if laryngeal symptoms, pertinently breathlessness and cough, are interpreted as manifestations of COVID‐19 or underlying disease progression. This might be particularly challenging for those with comorbid progressive respiratory disease, for example, chronic obstructive pulmonary disease (COPD). Psychosocially, symptoms of chronic cough may receive negative attention thus affecting well‐being. SLTs might also see a rise in psychogenic voice disorders. These could feasibly arise in response to direct COVID‐19 experiences; or because of difficulties dealing with uncertainties of COVID‐19 which are likely affecting all aspects of life.

Conversely, it is feasible that lockdown restrictions will positively influence laryngeal function for existing patients who developed dysphonia through high occupational vocal demands, owing to opportunities for voice rest if unable to attend work. For such patients, social and occupational restrictions may also provide opportunity to engage more actively in rehabilitation programmes.

#### Treatment implications

Face‐to‐face therapy delivery is the cornerstone of the evidence for dysphonia treatment. Routine out‐patient treatment programmes are unlikely to be an option for some time and as has been previously stated adaptations towards telehealth practice are inevitable. Perceptual assessment as a response to treatment trials is an essential tool yet is challenging in a virtual delivery mode. The quality and type of microphone are key for detailed capture of voice, and laryngeal function (Ward *et al*. 2014). An acoustic speech processor may be useful for measures of loudness, pitch and duration data (Weidner and Lowman 2020); and controlling noise and microphone position can reduce random error (Jannetts *et al*. 2019). Perceptual evaluation of upper body tension, through video‐consultation may also enhance treatment. Distinct telehealth challenges may arise for patients with moderate to severe dysphonia, notably those with breathiness and asthenia.

### Airway services

Patients with airway issues have been recommended to shield themselves, similarly to other vulnerable groups (Public Health England 2020). Complex airway patients are likely to be more susceptible to serious illness and negative consequences of treatment if they are infected with COVID‐19. The clinical rationale for this is threefold. First, patients who have undergone airway reconstruction procedures such as laryngotracheal reconstruction (LTR) or cricotracheal resection (CTR) are high risk and challenging to intubate (Crawley and Dalton 2015). Second, prolonged ventilation of patients with an altered airway is likely to lead to worse long‐term outcomes (e.g., further airway damage). Third, patients with complex airway disorders often experience difficulties with cough strength and secretion clearance (Tanner *et al*. 2019).

#### Assessment and monitoring

Rigorous triage is now necessary to identify the highest risk patients for urgent procedures. Before COVID‐19, endoscopy and videofluoroscopy were key adjuncts in the voice and swallowing management of airways patients (Clunie *et al*. 2017). However, the loss of instrumentation is an opportunity to showcase the range of skills SLTs use to evaluate and treat swallowing and voice difficulties with airways patients, as well as continuing to work closely with the broader MDT to form a clear, well‐evidenced treatment plan for complex airway patients. However, extended waiting lists and subsequent increased patient anxiety because of treatment delays is inevitable.

Attendance at highly specialist airway service clinics frequently involve expensive, long‐distance travel, which can be burdensome for people requiring specialist medical care. Telehealth is an opportunity to relieve this burden for complex airway patients. Similar to the extended ENT SLT roles described above, there is also an opportunity for developing SLT‐led surveillance clinics for complex airway patients. These allow ENT SLTs to use their skills to monitor swallowing, voice and airway problems remotely, and triage patients to ENT, nursing and psychology colleagues as required. This also has benefits for patients as there is no unnecessary repetition within their care pathway.

#### Long‐term impact

Clinical consensus indicates that COVID‐19 has the potential to cause airway damage as a result of laryngeal manifestations of the disease (Tysome and Bhutta 2020); as well as expected long‐term airway damage due to prolonged intubation and need for tracheostomy (Brodsky *et al*. 2018). As a result, airway services may see an increase in referrals in the coming months as a direct result of the virus itself. This is in addition to the number of existing patients whose treatment has been delayed in the initial response to the pandemic. Airways SLTs will need to prepare for this potential increase with forward workforce planning and upskilling colleagues to assist with management of patients where appropriate.

A summary of the main threats and opportunities for ENT SLT clinical services is provided in table 1.

**Table 1:** Threats and opportunities for HNC, voice and airways SLT services

	Main threats	Main opportunities
All ENT/SLT	Increased workload and patient complexity related to COVID‐19 voice and airway problems and delayed HNC diagnosis Reduced access to key instrumental procedures, e.g., FEES, endoscopy, VF Inequitable care, affecting patients and services without access to digital resources	New modes of service delivery such as telehealth/proxy consultation/multidisciplinary and cross‐service collaborations Increased skills and extended role: speech and language therapy (SLT)‐led assessment clinics and increased scope of practice Harnessing SLT expertise in perceptual assessment and non‐instrumental assessments
Head and neck cancer (HNC)	Reduced opportunity for SLT input to multidisciplinary team (MDT) meetings Challenges of providing face‐to‐face consultations with high‐risk groups in particular, e.g., laryngectomy Lack of timely rehabilitation after oncology treatment may lead to poorer long‐term outcomes	Patient access to centralized specialist SLT services via new modes of delivery Promotion of self‐management where feasible
Voice and airways	Delayed or inaccurate diagnoses in absence of laryngoscopy. Inappropriate treatment, and/or escalating symptom severity Unknown prevalence of COVID‐19‐related laryngeal disorders	Increased profile of SLT for management and rehabilitation of communication and upper airway disorders

Note: FEES, Flexible Endoscopic Evaluation of Swallowing; VF, Videofluoroscopy.

## Workforce issues

COVID‐19 presents members of the SLT profession with several workforce‐related challenges including redeployment of staff, capacity issues related to COVID‐19 staff sickness and increased demand with new and emerging caseloads at a time of national emergency. The recent Interim NHS People Plan (NHS Improvement 2019) recognizes that SLTs have broad ranging skills, deliver high‐quality care, limit unnecessary care costs and are key in reducing reliance on hospitals. Such workforce profiling and analysis suggests that the SLT workforce is therefore well positioned to develop and implement new strategies to deliver care during and after the COVID‐19 pandemic. Indeed, SLTs have already rapidly adapted to the changing COVID‐19 healthcare landscape which has mandated a shift in healthcare provision such as the shift towards telehealth solutions described above.

### Changes in workforce

As part of the national effort to manage COVID‐19, the Chief Allied Health Professionals in the UK, Health and Care Professions Council (HCPC), Council of Deans and Allied Health Professions Council have asked practitioners to be flexible and adopt new ways of working (NHS England 2020). To facilitate practitioners in the transition of potential new roles the governing bodies further specify provider organizations must be supportive of new ways of working. Such changes in working will be a departure from usual SLT practice, but despite this, SLTs are urged to follow principles of best practice, following HCPC guidance, and to use professional judgement to assess risk. As well as existing staff, there has been a call to former healthcare practitioners to return to the frontline workforce with expedited HCPC and RCSLT re‐registration processes in place. Students in advanced stages of their studies are also being offered an opportunity to join a temporary COVID‐19 register (RCSLT 2020a).

### Redeployment and meeting existing and emerging needs

The RCSLT has fully supported the redeployment of SLTs to support the health and social care system (RCSLT 2020a). However, services need to continue to support existing caseloads and new patients requiring input for communication and swallowing impairments. Any plans for the redeployment of SLTs must take account of prioritized services, that is, those that cannot be suspended due to the high risks involved if they did not continue. SLTs can play a key role in the prevention of hospital admissions and readmissions for vulnerable groups in the community. Further, SLTs assist in expediting inpatient discharge. Such contributions support the government's plan to shield the high risk and support wider risk groups (RCSLT 2020a).

The RCSLT has also highlighted the key skills and expertise which SLTs have to meet some of the clinical presentation needs of patients with COVID‐19 (RCSLT 2020a). SLTs in many centres are being redeployed, for example, to ICUs in ‘buddy’ roles to support nursing and medical staff managing acutely ill COVID‐19 patients. In addition to increased capacity, buddies are being used to support pressures on service provision due to increased COVID and non‐COVID‐related staff sickness. The ability to undertake new roles should be underpinned by access to appropriate training to prepare individuals (RCSLT 2020a), and potentially the opportunity to be upskilled to allow for continued professional development.

It is also emerging that those recovering from COVID‐19 are requiring the specialist skills of SLTs post‐COVID‐19 infection. In the ICU setting, if the patient is intubated but awake, the SLT can support communication including consent regarding treatment decisions using alternative and augmentative communication options. When patients are extubated and moving to intermediate‐level care, the SLT has an essential role in the supporting communication, swallowing and airway management (RCSLT 2020a). For patients who are receiving end‐of‐life care, SLTs can play a key role in advocacy and supporting quality‐of‐life‐focused decisions with regards to eating and drinking. Finally, SLTs can support the consent process for recruitment to local and national COVID‐19 clinical research trials.

### Health and well‐being

At a time of heightened pressure on SLTs, consideration needs to be given to COVID‐19 and non‐COVID‐19‐related health burden. Given the rapid pace of change in the healthcare landscape, usual consultation processes with staff are not possible. The RCSLT has provided a rapid response in producing guidance for the profession, including safety indications for PPE and aerosol‐generating procedures, applicable to ENT SLTs (Bolton *et al*. 2020).

Given the uncertainty and anxieties that staff may experience, NHS Employers (2020) have set out guidance for leaders highlighting the importance of regular and open communication. As well as physical health, increased efforts have been made to address mental health proactively with guidance for managers and several services being offered by companies for free, for example, Big Health, Headspace and Unmind (British Psychological Society 2020).

### Planning for the future of the workforce

The future remains uncertain, with the potential for further COVID‐19 case surges. However, healthcare providers are already considering post‐COVID‐19 service recovery and response plans. Healthcare organizations will need to recognize new models of good practice. As a part of this, workforce issues will be a critical element for consideration. There may be shifts from acute to community provision of services. Similarly, the rapid transition from traditional, face‐to‐face clinical service delivery to telehealth as described above may well become embedded. However, some services may not have adequate video‐conferencing resources and expertise, delaying access and increasing inequalities in service provision. Likewise, the increased use of information technology (IT) and virtual platforms for meetings and sharing resources may facilitate ongoing collaborative links across large geographical areas with minimal travel or venue hire costs. This may mean SLTs working remotely and making use of IT solutions which have been rolled out to facilitate this.

There is now a staged return to SLT‐led endoscopy services (RCSLT 2020d). Consideration will be needed for compliance with these guidelines and how this affects current caseloads, regardless of COVID status. This will be particularly challenging for training new SLT endoscopists. Extended practice roles such as triage systems and ENT SLT‐led Two Week Wait clinics will require further training, greater numbers of specialist skilled staff and equipment resources. The main threats and opportunities related to ENT/SLT workforce are summarized in table 2.

**Table 2:** Main threats and opportunities for the speech and language therapy (SLT) workforce in ear, nose and throat (ENT)/laryngology specialty areas

Main threats	Main opportunities
• Hands on training in endoscopic evaluation of the larynx (EEL), FEES and SVR • Redeployment and impact on existing services • Psychological impact on the SLT workforce	• Service innovation and models of care • Extended ENT/SLT practice roles • Implementation of telehealth services

Note: FEES, Flexible Endoscopic Evaluation of Swallowing; SVR, Surgical Voice Restoration.

## Leadership and research

### Leadership

NIHR CRN ENT SLT group members have expertise in generating, implementing and disseminating evidence‐based practice while remaining patient facing. They have extensive clinical research networks to ensure that their contributions to strategic direction on issues affecting ENT SLT delivery of care. The group has a shared focus and provides a forum for collective learning, innovation and research prioritization. It works across geographical and service boundaries and is driving high‐quality research that is clinically relevant and implementable across services. As described above, clinical issues and service delivery have altered at a fast pace, with little time to evaluate the evidence for such changes. This period of instability and change in priority‐focus requires a rethink of the ENT SLT collective research strategy.

### National research priorities

The ENT SLT research profile across the UK has several multidisciplinary, multi‐ and single‐centre studies open to recruitment and follow‐up (e.g., PATHOS CRUK/13/025 CPMS 18645; DARS CRUK/14/014 CPMS 19934; PITSTOP CPMS 39932) supported by the NIHR Portfolio. In addition, there are many studies led by ENT SLTs, funded by the NIHR Integrated Clinical Academic Programme as well as charitable funding. Currently, the NIHR, Medical and Healthcare products Regulatory Agency (MHRA), and European Medicines Agency (EMA) have prioritized nationally sponsored COVID‐19 studies and delivery of urgent public health research, vaccines and medicines (NIHR 2020, MHRA 2020, EMA 2020). The NHS Health Research Agency (HRA) has enabled a fast‐track ethical‐approval process to expediate study set‐up and provided advice on information and consent procedures for emergency research projects (NHS HRA 2020). NIHR clinical academic staff are advised to return to frontline care as directed by their employer. This means pausing ‘live’ studies and halting those in preparation. However, some host organizations have agreed that non‐COVID studies may continue, particularly where stopping a study may impact on patient care.

### ENT SLT research

The pandemic has slowed the progress of research into non‐COVID‐19 topics, created major disruption for ENT SLT‐related studies and arrested NIHR clinical fellowships. Common methodologies and methods used in ENT SLT research have been challenged and will continue to be in the post‐COVID era. For example, studies conducting instrumental tests are now severely restricted and their reintroduction into clinical, let alone research, practice is unclear, but likely to be slow. Furthermore, SLT research is frequently ‘patient‐facing’ and multidisciplinary. Commonly used methods such as focus groups and co‐design, with face‐to‐face contact for non‐essential activities, and in particular group gatherings will be restricted for some time. Again, these activities may continue via telephone or video‐conferencing, while recognizing potential exclusion of those unable or unwilling to access IT. Access to vulnerable patient groups, for example, older adults, people with a laryngectomy or tracheostomy will be further limited for an extended, undefined period of time. Conducting patient and public involvement events to establish research priorities and collaboration will be challenging. Furthermore, opportunities to disseminate research have reduced, with cancellation of international and national face‐to‐face meetings although some have transferred to virtual conference platforms.

### Funding

Many UK research councils are redirecting or repurposing funding to enable new treatments, diagnostics and vaccines to contribute to the understanding of, and response to, the COVID‐19 pandemic and its impacts (UK Research and Innovation 2020). The NIHR have now opened a funding call for research into COVID‐19 pandemic beyond the acute phase (NIHR 2020b). Charitable research funders have experienced significant downturn in their income necessitating an immediate cut in research spending. For example, Cancer Research UK (CRUK) predict this will set back the UK cancer research programme, potentially for many years, and are postponing funding decisions for the majority of 2020.

### Research opportunities

RCSLT Clinical Excellence Networks and the NIHR CRN ENT SLT Research Group have been essential to information dissemination, best practice and problem‐solving. Agreement on prospective data collection to evaluate altered service models is an imperative. Limited learning will occur if this work is conducted uni‐professionally, in silos with small single‐centre data collection. National databases have been set up, including the RCSLT COVID‐19 and Head and Neck Oncology CEN Laryngectomy data set, ENT‐UK National COVID‐19 Tracheostomy Service Evaluation to investigate voice and swallowing outcomes and changes to service provision.

Now more than ever, there is a need to upskill to generate and evaluate the best evidence to respond to this changed landscape. The NIHR recognizes the importance of maintaining a research pipeline and their funding and training schemes will remain open, with some changes to application deadlines The NIHR CRN ENT SLT group have met to discuss research priorities for clinical considerations and service delivery. Examples of these priorities include:

- Understanding post‐extubation laryngeal trauma, its impact, trajectory for recovery and management of associated voice and swallowing problems.
- Identifying risk factors for dysphagia following tracheostomy placement and effective dysphagia rehabilitation.
- Understanding the respiratory–swallow cycle, cough and laryngeal function in patients with acute and chronic respiratory disease.
- Reliability and validity of non‐invasive voice and swallowing screening and assessment tools, including those that can be conducted remotely.
- Collection of standardized outcome measures, using digital technologies where possible.
- Impact of altered HNC treatment schedules on functional outcome.

Service delivery and models of care need evaluation and further development as to how we can best support patients, the workforce and the NHS as a whole. Examples of priorities include:

- Provision of remote highly specialist care for our most vulnerable patients.
- Extent of AGP for routine ENT SLT procedures and how they are best managed.
- Models of telehealth for ENT SLT services.
- Implementation of ENT SLT‐led clinics and triaging.
- The main threats and opportunities to ENT/SLT research are summarized in table 3.

**Table 3:** Main threats and opportunities for speech and language therapy (SLT) clinical research in ear, nose and throat (ENT)/laryngology

Main threats	Main opportunities
• Reduction in funding calls and high competition • Current research affected by access to participants and ability to conduct face‐to‐face and instrumental assessments	• Greater collaboration across ENT SLT group • Changes in service delivery warranting further investigation • Generation of national databases • New set of research priorities

## Discussion

The aim of this paper was to provide an expert discussion on the threats and opportunities from the COVID‐19 pandemic for SLTs in ENT/laryngology practice. The NIHR CRN ENT SLT Research Group represents a collaboration of experienced clinical researchers in ENT/laryngology with a broad geographical representation across England.

It is clear that the COVID‐19 pandemic has had and will continue to have a significant impact on all aspects of SLT practice and research in ENT/laryngology (HNC, voice and airway disorders). The implications of the current situation with respect to the ENT/laryngology clinical area are significant, but with recognition of there is considerable overlap with SLT services in respiratory care, critical care and dysphagia care generally. It is also recognized that there are several major issues that require evaluation for the whole of the NHS workforce (e.g., PPE provision and mental health and well‐being) (Sayburn 2020, Unadkat and Farquhar 2020). Whilst these issues are highly relevant to many SLTs, the issues are not profession specific and consequently mentioned only briefly here.

It is evident that the immediate impact of the pandemic has resulted in major disruption to all aspects of clinical delivery, workforce and research. It remains unclear as to when any of these areas will resume operations and if the post‐COVID era will have changed clinical practice, professional remits and research priorities forever. Clearly a significant opportunity exists in the post‐COVID era to re‐evaluate current practice, embrace and evaluate new ways of working, requiring strategic planning, coordination, collaboration, and dissemination across the UK.
